# Arteriovenous fistula as adjuvant treatment in arterial revascularization of an at-risk limb

**DOI:** 10.1590/1677-5449.210042

**Published:** 2021-09-10

**Authors:** Júlio César Gomes Giusti, Sabrina Payne Tartarotti, Fabio Henrique Rossi, João Paulo Neves Beraldo, Francisco Cardoso Brochado

**Affiliations:** 1 Hospital Municipal do Tatuapé Dr. Carmino Caricchio (HMCC), São Paulo, SP, Brasil.; 2 Instituto Dante Pazzanese de Cardiologia (IDPC), São Paulo, SP, Brasil.

**Keywords:** vascular graft, arteriovenous fistula, peripheral arterial disease, skin transplantation

## Abstract

Acute arterial occlusion remains a major challenge for the vascular surgeon. The therapeutic approach depends mostly upon the severity of the tissue injury and the duration of symptoms. Several techniques are available in the current therapeutic arsenal, however, regardless of the technique chosen, postoperative factors frequently observed, such as poor outflow status, or even low graft flow, can contribute negatively to the outcome of revascularization. We describe a case of acute limb ischemia, in the postoperative period of a femoral-tibial bypass, which was occluded due to outflow limitation and high peripheral vascular resistance. The patient underwent a second tibial revascularization combined with construction of an arteriovenous fistula, followed by forefoot amputation and partial skin graft. An energetic approach to the at-risk limb makes it possible to reduce unfavorable outcomes, such as amputation and death, and accelerates recovery of tissues affected by acute ischemia.

## INTRODUCTION

Acute arterial occlusion (AAO) is a consequence of a sudden obstruction of blood flow, usually related to arterial emboli or thrombosis. The abrupt interruption of the supply of blood and nutrients to metabolically active tissues, such as nerves, muscles, and skin, frequently results in a critical state of limb ischemia.^1^^,^^2^ Consequently, when AAO is not recognized and treated promptly, it can cause irreversible tissue damage, loss of the limb, and death.^1^ The incidence of AAO is approximately 1.5 cases/10,000 person-years and around 10 to 15% of cases require a major amputation while in hospital.^2^

The treatment approach depends on the severity of the tissue damage and the duration of the symptoms. In patients with acute cases, in which viability of the limb is maintained, the objective of treatment is to restore the blood flow and reverse the severe ischemic state.^1^^-^3 Nowadays, several techniques are available, the most commonly used of which are thromboembolectomy, thrombolytic therapies, endovascular recanalization, and arterial bypass.^1^^,^^4^ However, regardless of the technique chosen, factors such as limited arterial outflow and low flow through arterial grafts that are frequently observed in AAO can have significant impact on the patency of the revascularization and, consequently, on limb salvage.^5^^-^7

Construction of an arteriovenous fistula (AVF) combined with arterial revascularization has been employed for decades^5^^-^8 as an adjuvant procedure with the objective of reducing peripheral vascular resistance (PVR) and increasing blood flow through the revascularized segment as a consequence.^6^^,^^9^

Free and informed consent was obtained and the study was approved by the institutional Ethics Committee (ruling nº 4.716.113).

## PART I – CLINICAL SITUATION

**Figure 1:**
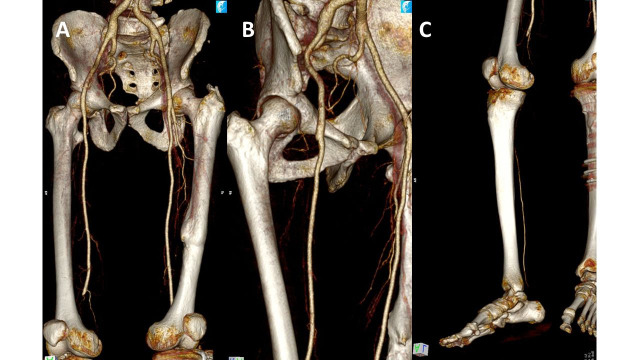
**(A)** Computed tomography angiography of the lower limbs showing occlusion of the popliteal segment; **(B)** deep femoral artery, and **(C)** the tibioperoneal trunk, with segmental refilling of the posterior tibial artery.

The patient was a 51-year-old white male, smoker, crack user, and alcoholic, who presented at our service with a history of pain in the right plantar region with sudden onset 15 days previously that had progressed to cyanosis of the toes and sharp pains in the limb. During the physical examination, irreversible cyanosis and blistering were observed involving the entire forefoot, with restricted dorsiflexion. On the right-hand side, palpation only detected a 3/3+ femoral pulse and flow was absent on Doppler imaging in inframalleolar arteries. The diagnostic hypothesis raised was Rutherford IIB AAO and an arterial study with contrast was requested. Computed tomography angiography (angio-CT) detected occlusion of the deep femoral artery (DFA) and popliteal artery, with segmental refilling of the posterior tibial artery only (Figure 1).

**Figure 2:**
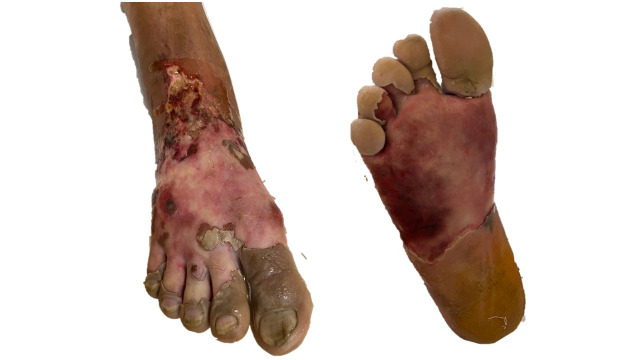
Appearance of the right foot on the 1st postoperative day after femoral-tibial bypass, after debridement of blistered tissue.

Thromboembolectomy of the DFA was performed immediately, followed by construction of an arterial bypass from the superficial femoral to the posterior tibial, using the ipsilateral great saphenous vein *ex situ*, devalved and not inverted. The following day, the patient’s pain had worsened, his creatine phosphokinase (CPK) levels had risen, and the bypass had occluded (Figure 2).

In view of this status, the following treatment options were discussed: 1) amputation at the thigh (transfemoral); 2) endovascular recanalization of popliteal arteries, tibiofibular trunk and plantar arch; or 3) previous bypass graft revision and construction of a plantar AVF.

## PART II – WHAT WAS DONE

We decided to perform thromboembolectomy of the bypass graft and construct an AVF between the common plantar artery and vein, with the objective of reducing PVR. The procedure was performed with spinal anesthesia and the medical team employed magnification with surgical loupes.

**Figure 3:**
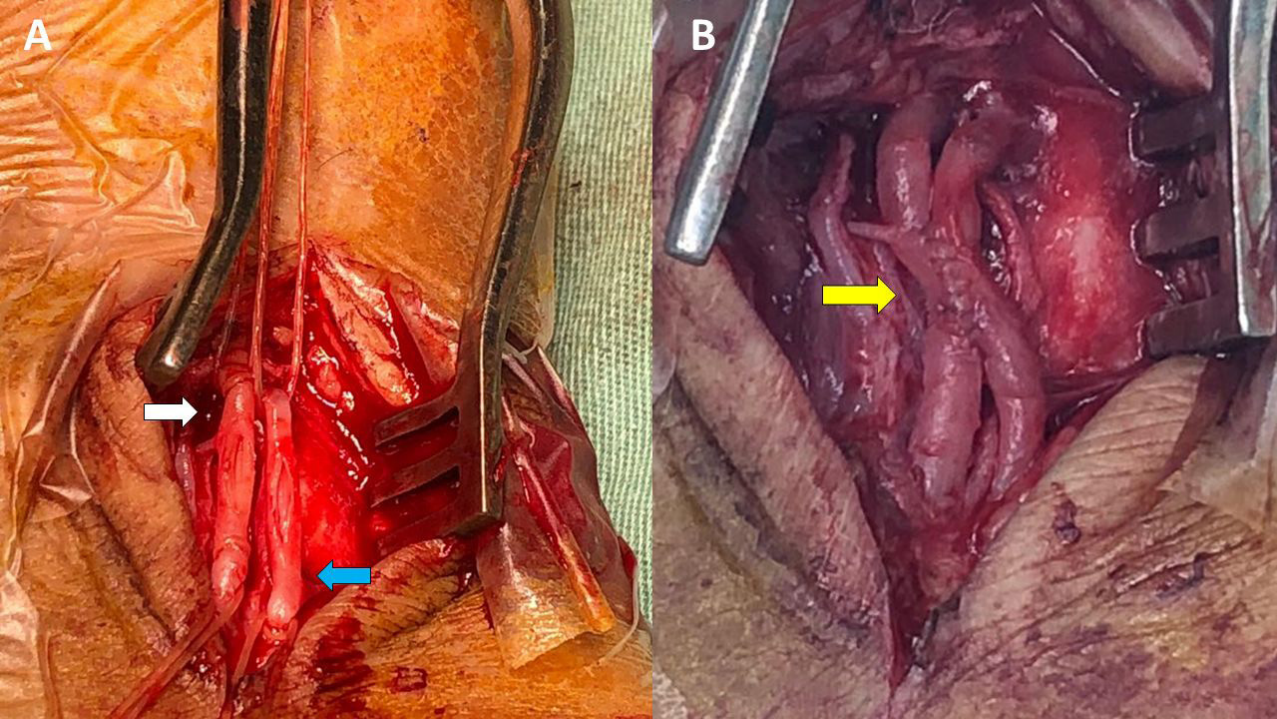
Images showing construction of the arteriovenous fistula between the common plantar artery and vein. **(A)** common plantar artery (white arrow) and common plantar vein (blue arrow); **(B)** Final result after construction of the arteriovenous fistula (yellow arrow).

During the operation, the graft was removed from the tunnel that had been created previously and thromboembolectomy was accomplished without complications, resulting in pulsating flow through the substitute vessel. After heparinization of the graft, it was replaced back in the tunnel and distal anastomosis was performed again, using a single continuous 7-0 polypropylene suture and an end-to-side technique. A longitudinal 5 cm incision was made in the skin over the common plantar artery and both segments (arterial and venous) of the plantar bundle were located (Figure 3A). The common plantar artery was pulsatile and fibroelastic. The AVF was constructed with a side-to-side technique using a single 7-0 polypropylene suture, resulting in palpable thrill (Figure 3B).

**Figure 4:**
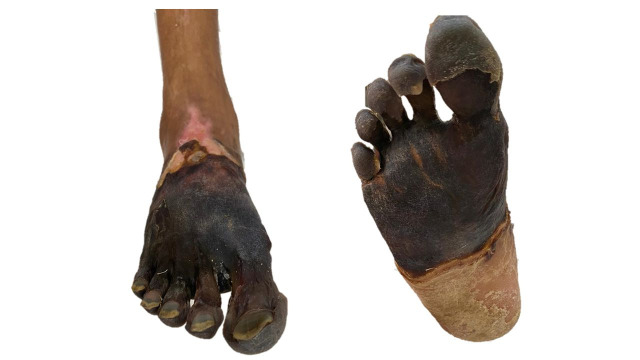
Dry, well-delineated, necrosis of the right forefoot.

**Figure 5:**
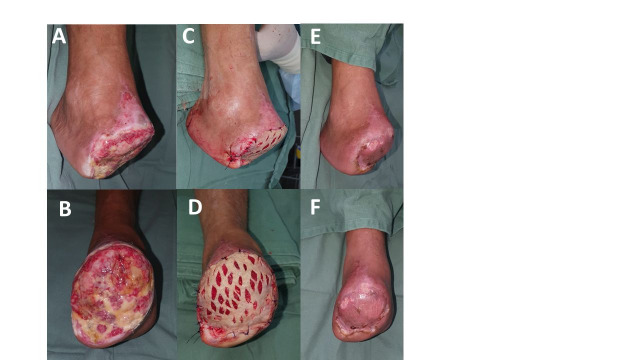
**(A** and **B)** 10th day after transmetatarsal amputation of the right foot; **(C** and **D)** Immediate postoperative period after the skin autograft, performed by the reconstructive plastic surgery team at the Hospital Municipal do Tatuapé Dr. Carmino Caricchio (HMCC), Brazil; **(E** and **(F)** Fully healed amputation bed, at outpatients consultation, 48 days after the autograft.

During the postoperative period, the patient reported substantial improvement of pain and his thermal gradient reduced. A posterior tibial pulse was palpable (3/3+). While still in hospital (20th postoperative day), the forefoot necrosis became delimited (Figure 4) and the elevated CPK levels reduced and the patient was scheduled for transmetatarsal amputation (Figures 5A and 5B). After preparation of the amputation bed, a skin autograft was performed by the reconstructive plastic surgery team (Figures 5C and 5D). The patient was discharged from hospital and is in outpatients follow-up, with a fully healed amputation bed (Figures 5E and 5F) and is able to walk with the aid of ortheses. Secondary patency was 151 days.

## DISCUSSION

Limb loss rates due to AAO can be as high as 30% within the first month after presentation with ischemia and 1-year mortality is approximately 20%, making this condition a major challenge for vascular surgeons.^4^ Moreover, when compared with elective arterial surgery, patients revascularized because of AAO have an almost 20% higher risk of perioperative adverse events and double the likelihood of acute myocardial infarction.^10^

Countless therapeutic approaches are available for employment in immediate revascularization of the ischemic limb. These range from endovascular techniques, usually with thrombolytic therapy, to surgical procedures such as thromboembolectomy, arterial bypass, and primary amputation.^2^^-^4^,^^10^ Management of acute limb ischemia often involves a combination of operating techniques, aiming to restore blood flow and preserve viability of the tissues involved.^1^^-^3 One factor that limits salvage of limbs affected by AAO is high PVR in conjunction with low arterial flow. This concept was proposed decades ago by Sauvage et al.^11^ and popularized by Ascer et al.,^8^ who described construction of an AVF as an adjuvant to distal anastomosis of prosthetic below-the-knee grafts, with the objective of increasing the flow across the graft by reducing PVR. The limited outflow contributes aggressively to revascularization failure and is usually related to loss of patency and major amputation.^5^^,^^6^

In the case presented here, the decision to construct an arterial bypass was prompted by the infragenicular arterial involvement and the delay since onset of symptoms, which limited the results of any attempt to perform thromboembolectomy or thrombolysis of the crural arteries. In a similar manner, Creager et al.^2^ reported their preference for arterial bypass in cases with onset of symptoms more than 14 days previously. The technique of devalving the arterial substitute, under pulsating flow, is preferred at our service because it preserves the anatomic configuration of the native arteries in terms of their proximal and distal diameters, enabling more compatible anastomoses, in addition to maintaining arterial phasicity through the vessel.^12^^,^^13^ The absence of inframalleolar arteries limited flow to the foot and probably caused occlusion of the original revascularization. Therefore, the hypothesis of high arterial resistance in the limb was considered and the decision was taken to construct an AVF, which was essential to reduce the PVR and provoke increased blood flow through the graft, thereby averting rethrombosis.

Laurila et al.^5^ compared infrapopliteal arterial grafts for treatment of critical ischemia in groups with and without adjuvant AVF, demonstrating a significant increase in arterial flow measured in the substitute vessel in the group in which an AVF was constructed (p = 0.003). In the safety analysis, mortality and perioperative complication rates were similar in both groups, demonstrating that the technique did not increase morbidity or mortality.

In contrast, Aherne et al.^6^ recently conducted a meta-analysis of nine eligible studies with 408 arterial bypasses for treatment of critical ischemia, in 203 of which an adjuvant AVF was constructed, and concluded that there were no statistical differences in terms of patency, limb salvage, or perioperative morbidity and mortality. Although these authors did not observe data that support adjuvant AVF construction, the quality of the available scientific evidence remains limited, reflecting the scarcity of publications and the low number of randomized studies.^5^^,^^6^^,^^8^^,^^9^^,^^14^^,^^15^

The partial skin autograft technique has been in use for years in vascular surgery, usually performed by multidisciplinary teams that manage patients with peripheral arterial disease.^16^^-^19 The main objectives are to accelerate healing, reduce pain and manipulation of wounds, attenuate expenditure on synthetic coverings, and rapidly rehabilitate the patient.^16^^,^^17^ In the case presented here, the autologous covering enabled the extensive forefoot amputation to heal over, reducing the need for dressings by months. Naz et al.^16^ demonstrated that partial skin autografts in this group of revascularized patients not only afforded a durable and stable covering, but also reduced complications related to wounds and dressings and even improved limb salvage rates.

Once possible, criticism of our initial approach would be the possibility of constructing an AVF during the first revascularization or attempting catheter-guided fibrinolysis with the objective of improving outflow below the knee. Additionally, another adjuvant option to save the graft could have been distal inframalleolar recanalization by percutaneous transluminal angioplasty. However, appropriate endovascular materials for these interventions were not available when the patient was admitted.

Revascularization by arterial bypass remains an important tool for limb salvage in patients with AAO. Adjuvant treatments, such as construction of an AVF, can help to increase graft patency and, consequently, to save the limb. Additionally, skin autografts considerably shorten the length of hospital stay and speed up wound healing, helping to control pain, and reducing expenditure on dressings. Therefore, management of the at-risk limb using multiple surgical strategies with a multidisciplinary approach can reduce unfavorable outcomes related to AAO, such as amputations and deaths, and accelerate recovery of tissues affected by acute ischemia.
