# Supplementary material for: Arteriovenous fistula as adjuvant treatment in arterial revascularization of an at-risk limb
Source: J Vasc Bras. 2021 Sep 10;20:e20210042. doi: 10.1590/1677-5449.210042 (PMC9045530; doi:10.1590/1677-5449.210042)
Supplement: Translation

# Translation: Fístula arteriovenosa como tratamento adjunto na revascularização arterial do membro em risco

**Authors:** Júlio César Gomes Giusti, Sabrina Payne Tartarotti, Fabio Henrique Rossi, João Paulo Neves Beraldo, Francisco Cardoso Brochado

**Affiliations:** 1 Hospital Municipal do Tatuapé Dr. Carmino Caricchio (HMCC), São Paulo, SP, Brasil.; 2 Instituto Dante Pazzanese de Cardiologia (IDPC), São Paulo, SP, Brasil.

**Keywords:** enxerto vascular, fístula arteriovenosa, doença arterial periférica, transplante de pele

## Abstract

A oclusão arterial aguda do membro inferior continua sendo um grande desafio para o cirurgião vascular. A abordagem cirúrgica depende principalmente da gravidade da lesão tecidual e da duração dos sintomas. Diversas técnicas estão disponíveis no arsenal terapêutico atual; porém, independentemente da técnica escolhida, fatores pós-operatórios, como o escoamento arterial limitado e o baixo fluxo nos substitutos arteriais, podem contribuir negativamente no resultado da revascularização. Descrevemos um caso de oclusão arterial aguda de membro inferior, no pós-operatório de uma derivação femorotibial, que se encontrava ocluída devido a limitação de escoamento e a alta resistência vascular periférica. Foi submetido a nova revascularização femorotibial, associada à confecção de uma fístula arteriovenosa, seguido de amputação de antepé e enxerto parcial de pele. O investimento enérgico no membro em risco possibilita reduzir os desfechos desfavoráveis, como amputação e óbito, e acelera a recuperação dos tecidos acometidos pela isquemia aguda.

## INTRODUÇÃO

A oclusão arterial aguda (OAA) é consequência da obstrução repentina do fluxo sanguíneo, usualmente relacionada a trombose ou embolia arterial. A abrupta cessação de suporte sanguíneo e nutrientes ao tecido metabolicamente ativo, como nervos, músculos e pele, resulta frequentemente em um quadro crítico de isquemia do membro^1^^,^^2^. Consequentemente, quando a OAA não é prontamente reconhecida e tratada, pode acarretar lesão tecidual irreversível, inviabilidade do membro e morte^1^. A incidência da OAA é de aproximadamente 1,5 casos/10.000 pessoas-ano, sendo que 10 a 15% dos acometidos evoluem para amputação maior ainda durante a internação^2^.

A abordagem terapêutica depende da gravidade da lesão tecidual e da duração dos sintomas. Para aqueles com quadro agudo, que preservam a viabilidade do membro, o tratamento tem por objetivo restaurar o fluxo sanguíneo revertendo a grave isquemia instaurada^1^^-^3. Atualmente, diversas técnicas estão disponíveis, sendo as mais comumente utilizadas a tromboembolectomia, as terapias trombolíticas, a recanalização endovascular e a derivação arterial^1^^,^^4^. Porém, independentemente da técnica escolhida, fatores frequentemente observados na OAA, como o escoamento arterial limitado e o baixo fluxo nos substitutos arteriais, podem interferir significativamente na perviedade da revascularização e consequentemente no salvamento do membro^5^^-^7.

A criação da fístula arteriovenosa (FAV) associada à revascularização arterial tem sido utilizada há décadas^5^^-^8 como procedimento adjunto e tem como objetivos a redução da resistência vascular periférica (RVP) e o consequente aumento do fluxo sanguíneo no segmento revascularizado^6^^,^^9^. O termo de consentimento livre e esclarecido foi obtido, e o estudo foi aprovado pelo Comitê de Ética da instituição (parecer nº 4.716.113).

## PARTE I - SITUAÇÃO CLÍNICA

O paciente era um homem branco, 51 anos, tabagista, usuário de crack e etilista, o qual deu entrada em nosso serviço com história de dor súbita em região plantar direita há 15 dias, evoluindo com cianose de pododáctilos e dor lancinante no membro. Ao exame físico, observou-se cianose fixa e flictenas acometendo todo o antepé, associada a redução da dorsiflexão. A palpação evidenciou somente pulso femoral 3/3+ a direita, e notou-se ausência de fluxo (Doppler) em artérias inframaleolares. Foi realizada a hipótese diagnóstica de OAA Rutherford IIB, e optou-se por estudo contrastado. A angiotomografia computadorizada (angio-TC) detectou oclusão de artéria femoral profunda (AFP) e artéria poplítea, com reenchimento segmentar apenas da artéria tibial posterior (Figura 1).

**Figura 1:** **(A)** Angiotomografia computadorizada de membros inferiores evidenciando oclusão do segmento poplíteo; (**B)** da artéria femoral profunda e (**C)** do tronco tibiofibular, com reenchimento segmentar de artéria tibial posterior.

Prontamente, foi realizada a tromboembolectomia de AFP, seguida de uma derivação arterial da femoral superficial para tibial posterior, com veia safena magna ipsilateral *ex situ*, não invertida e devalvulada. No dia seguinte, o paciente evoluiu com piora da dor, elevação dos níveis de creatinofosfoquinase (CPK) e oclusão da derivação (Figura 2).

**Figura 2:** Aspecto do pé direito, no 1º dia de pós-operatório de derivação femorotibial, após desbridamento de flictenas.

Diante desse quadro, as seguintes opções terapêuticas foram discutidas: 1) amputação de coxa (transfemoral); 2) recanalização endovascular de artérias poplítea, tronco tibiofibular e arco plantar; ou 3) revisão de enxerto prévio e confecção de FAV plantar.

## PARTE II - O QUE FOI FEITO

Optamos pela tromboembolectomia de enxerto prévio e confecção de FAV entre a artéria e a veia plantares comum, com o objetivo de reduzir a RVP. O procedimento foi realizado sob raquianestesia, e a equipe médica utilizou magnificação através de lupas cirúrgicas.

No intraoperatório, o enxerto foi retirado do túnel previamente confeccionado, e a tromboembolectomia foi realizada sem intercorrências, resultando em fluxo pulsátil no substituto. Após heparinização do conduto, o enxerto foi tunelizado, e a anastomose distal foi novamente confeccionada com fio único de polipropileno 7-0, através de técnica terminolateral. Uma incisão longitudinal de pele, com 5 cm de extensão, foi realizada sobre a artéria plantar comum, e ambos os segmentos (arterial e venoso) do feixe plantar foram reparados (Figura 3A). A artéria plantar comum era pulsátil e apresentava aspecto fibroelástico. A FAV foi confeccionada em técnica laterolateral, com fio único de polipropileno 7-0, resultando em frêmito palpável (Figura 3B).

**Figura 3:** Imagem representando a confecção da fístula arteriovenosa entre a artéria e a veia plantares comum. (**A)** Artéria plantar comum (seta branca) e veia plantar comum (seta azul). (**B**) Resultado final após confecção de fístula arteriovenosa (seta amarela).

No pós-operatório, o paciente apresentou melhora substancial da dor e do gradiente térmico. Notou-se a presença de pulso tibial posterior (3/3+). Durante a internação (20º dia de pós-operatório), houve delimitação da necrose de antepé (Figura 4) e diminuição dos altos níveis de CPK, sendo, então, indicada amputação transtársica (Figura 5A e 5B). Após preparo do leito de amputação, optou-se pela autoenxertia de pele, realizada pela equipe da cirurgia plástica reparadora (Figura 5C e 5D). O paciente recebeu alta hospitalar e segue ambulatorialmente, com cicatrização completa de leito de amputação (Figura 5E e 5F) e preservação da deambulação sob auxílio de órtese. A perviedade secundária foi de 151 dias.

**Figura 4:** Necrose seca de antepé direito, bem delimitada.

**Figura 5:** (**A**) e (**B**) 10º dia de pós-operatório da amputação transtársica de pé direito. (**C**) e (**D**) Pós-operatório imediato da autoenxertia de pele realizada pela equipe de cirurgia plástica reparadora do Hospital Municipal do Tatuapé Dr. Carmino Caricchio (HMCC). (**E**) e (**F**) Cicatrização completa do leito de amputação, em consulta ambulatorial, após 48 dias da autoenxertia.

## DISCUSSÃO

A taxa de perda de membro por OAA pode chegar até 30% ainda no primeiro mês da apresentação do quadro isquêmico, com mortalidade de aproximadamente 20% no primeiro ano, o que faz dessa complicação um grande desafio para o cirurgião vascular^4^. Ademais, quando comparada às cirurgias arteriais eletivas, o paciente revascularizado por OAA apresenta quase 20% maior risco de eventos adversos perioperatórios e o dobro de chance de evoluir com infarto agudo do miocárdio^10^.

Inúmeras abordagens terapêuticas estão disponíveis para serem utilizadas na revascularização imediata do membro isquêmico, e incluem técnicas endovasculares, usualmente através de terapia trombolítica, assim como procedimentos cirúrgicos como a tromboembolectomia, a derivação arterial e a amputação primária^2^^-^4^,^^10^. Frequentemente, a abordagem da isquemia aguda de membro envolve a combinação de técnicas operatórias com o objetivo de restaurar o fluxo sanguíneo e preservar a viabilidade do tecido afetado^1^^-^3. Uma das conhecidas limitações no salvamento do membro acometido pela OAA é a elevada RVP associada ao baixo fluxo arterial. Esse conceito foi proposto há décadas por Sauvage et al.^11^ e difundido através de Ascer et al.^8^, que descreveram a confecção de uma FAV adjuvante à anastomose distal de enxertos infrageniculares protéticos, com o objetivo de aumentar o fluxo no conduto, através da redução da RVP. Essa limitação de escoamento contribui agressivamente para o insucesso da revascularização e, usualmente, está relacionada à perda de perviedade e amputação maior^5^^,^^6^.

No caso aqui apresentado, a opção pela derivação arterial foi motivada pelo acometimento das artérias infrageniculares associada à história prolongada do início dos sintomas, limitando, assim, o resultado de uma possível tromboembolectomia ou trombólise de artérias crurais. Creager et al.^2^, de forma similar, relataram preferência para a derivação arterial em casos com início dos sintomas há mais de 14 dias. A técnica de devalvulação do substituto arterial, sob fluxo pulsátil, é a preferência em nosso serviço, pois preserva o padrão anatômico das artérias nativas em relação aos seus diâmetros proximal e distal, permitindo anastomoses mais compatíveis, além de manter a fasicidade arterial no conduto^12^^,^^13^. A ausência de artérias inframaleolares limitava o escoamento para o pé, o que provavelmente causou a oclusão da revascularização inicial. Dessa forma, foi aventada a hipótese de alta resistência arterial no membro, e optou-se pela FAV, que foi fundamental para reduzir a RVP e propiciar um aumento de fluxo sanguíneo no enxerto, evitando, assim, a retrombose.

Laurila et al.^5^, ao comparar as derivações arteriais infrapoplíteas no tratamento da isquemia crítica em grupos com e sem adjuvância de FAV, demonstraram um aumento significativo do fluxo arterial aferido no substituto, no grupo submetido a confecção da FAV (p = 0,003). Na avaliação de segurança, a mortalidade e as complicações perioperatórias apresentaram taxas similares entre os grupos, demonstrando que a técnica não elevou a morbimortalidade.

Em contrapartida, Aherne et al.^6^, em metanálise recente com nove estudos elegíveis, analisaram 408 derivações arteriais para tratamento de isquemia crítica, sendo 203 submetidas a confecção adjuvante de FAV. Concluíram que não houve diferença estatística em termos de perviedade, salvamento de membro e morbimortalidade perioperatória. Apesar de os autores não terem observado dados que sustentem a confecção adjuvante da FAV, a qualidade da evidência científica disponível permanece limitada, refletindo a escassez de publicações e o número reduzido de trabalhos randomizados^5^^,^^6^^,^^8^^,^^9^^,^^14^^,^^15^.

A técnica de autoenxertia de pele parcial já é utilizada há anos pela cirurgia vascular, usualmente através de equipes multidisciplinares que acompanham o paciente portador de doença arterial periférica^16^^-^19. Os principais objetivos são acelerar a cicatrização, reduzir a dor e a manipulação das feridas, reduzir potenciais custos com coberturas sintéticas e reabilitar rapidamente o paciente^16^^,^^17^. No caso aqui apresentado, a cobertura autóloga permitiu a cicatrização da extensa amputação de antepé, reduzindo em meses a necessidade de curativo. Naz et al.^16^ demonstraram que a autoenxertia de pele parcial nesse grupo de pacientes revascularizados, além de propiciar uma cobertura durável e estável, reduziu as complicações relacionadas às feridas e aos curativos, auxiliando inclusive nas taxas de salvamento de membro.

Como crítica à nossa conduta inicial, poderíamos citar a possibilidade de realização da FAV já na primeira revascularização ou, ainda, a tentativa de fibrinólise direcionada via cateter com o intuito de melhorar o escoamento infragenicular. Além disso, como opção adjunta ao salvamento do enxerto, a recanalização distal inframaleolar através de angioplastia transluminal percutânea poderia ser aventada. Entretanto, não dispúnhamos de material endovascular adequado para essas intervenções no momento da internação.

A revascularização através da derivação arterial permanece sendo uma importante ferramenta para o salvamento de membro no paciente acometido pela OAA. Terapias adjuvantes como a confecção de FAV podem auxiliar no aumento da perviedade do enxerto e, consequentemente, na preservação do membro. Ademais, a autoenxertia de pele reduz consideravelmente o tempo de internação e de cicatrização de feridas, além de auxiliar no controle da dor e dos custos relacionados ao curativo. Portanto, o investimento no membro em risco através de múltiplas estratégias cirúrgicas concomitantes à abordagem multidisciplinar possibilita reduzir os desfechos desfavoráveis relacionados à OAA, como amputação e óbito, e acelera a recuperação dos tecidos acometidos pela isquemia aguda.
